# Examining the inaccurate reproductive health messaging of crisis pregnancy centers: A comparative qualitative study

**DOI:** 10.1371/journal.pone.0325740

**Published:** 2025-06-05

**Authors:** Evangeline Warren, Alexandra Kissling, Alison H. Norris, Priya R. Gursahaney, Maria F. Gallo

**Affiliations:** 1 Department of Sociology, The Ohio State University, Columbus, Ohio, United States of America; 2 Oracle Life Sciences, Austin, Texas, United States of America; 3 Population Research Center, University of Maryland, College Park, Maryland, United States of America; 4 Division of Epidemiology, College of Public Health, The Ohio State University, Columbus, Ohio, United States of America; 5 Department of Obstetrics & Gynecology, College of Medicine, University of Cincinnati, Cincinnati, Ohio, United States of America; 6 Department of Epidemiology, School of Global Public Health, University of North Carolina, Chapel Hill, North Carolina, United States of America; Tribhuvan University, NEPAL

## Abstract

**Objectives:**

Given the prevalence of inaccurate or misleading health messaging linked to crisis pregnancy centers (CPCs) in the literature, we sought to understand what role, if any, standardized training materials might play in perpetuating common themes in reproductive health misinformation.

**Study design:**

We identified inaccurate health messages present in qualitative interviews with 10 staff members from 8 CPCs in Ohio. Separately, we conducted a content analysis of training manuals from two parent organizations, Care Net and Heartbeat International, to understand the form and substance of inaccurate health information messages in official CPC training materials. We compared the content of the two data sources to identify themes common to both, representing both individual and institutional level dissemination of false health messages.

**Results:**

We found that parent organizations provide false and misleading information in their training materials, often presenting such information as factually true or accurate. In interviews with a self-identified researcher, CPC staff relayed similar inaccurate health messages about the dangers of abortion and contraception, efficacy of condoms, and causes of infertility, strongly endorsing this false information. As an incidental finding, we documented enthusiasm for dissemination of inaccurate health information by CPC staff members who engage in public outreach and education.

**Conclusions:**

We found clear similarities in the inaccurate health messages provided by staff to researchers and the official training documents published by the CPC parent organizations. Parent organizations, therefore, may represent a potent source of reproductive health misinformation.

## 1. Introduction

In recent years, health experts have grown increasingly concerned about the rise in, and new sources of, health misinformation [1–3]. For example, the COVID-19 pandemic made apparent both the dangers of vaccine misinformation and the ability of a small set of actors to proliferate misinformation on a large scale. Notably, twelve individuals were found to be connected to nearly two thirds of all anti-vaccine content [4]. Within the reproductive health landscape, discussion forums for new parents have amplified unsubstantiated or misleading claims about the risks of interventions during pregnancy, labor, and delivery [5,6], and crisis pregnancy center (CPC) websites have been identified as sources of online misinformation, particularly that related to abortion and contraception [7,8]. In the present study, we build upon existing work establishing the prevalence of reproductive health misinformation on CPC websites by examining training materials produced by CPC parent organizations to understand whether CPC parent organizations might be considered particularly potent sources of misinformation. We find that the reproductive health misinformation present in training materials bears striking similarities to the misinformation previously identified on CPC websites [7,8], in interactions with CPC staff [9], and in our own interviews with CPC staff and volunteers.

### 1.1 Reproductive health misinformation

The consumption of health misinformation directly impacts patient behavior and health outcomes [3]. Patients who seek out health information from unregulated sources like social media often demonstrate negative health behaviors such as vaccine refusal [10]. When considering *reproductive* health misinformation in particular, this association between exposure and behavior is particularly concerning. Research has documented the presence of reproductive health misinformation on social media and other unregulated sites regarding, for example, abortion [7,11], contraception [12], pregnancy [13], and fertility [14]. Patients seeking out reproductive health information may not share what they have learned with their providers, allowing misinformation to take hold unchecked in their decision making [15,16]. In state contexts where laws and regulations restrict reproductive healthcare access, sexual education, and sexual health resources, and in environments where abortion is stigmatized, patients may turn to informal, non-clinical resources to seek reproductive health care knowledge, which leaves them particularly susceptible to exposure to reproductive health misinformation [17]. This is a particularly salient risk for younger patients and patients of color who already demonstrate greater susceptibility to misinformation [18] and have high endorsement of state-mandated reproductive health misinformation [19].

### 1.2 Overview of crisis pregnancy centers

In the United States, crisis pregnancy centers (CPCs, also known as pregnancy resource centers) are an arm of the anti-abortion movement, with contemporary anti-abortion advocates positioning CPCs as alternatives to existing medical and abortion clinics in communities [20]. Individual CPCs often are affiliated with international umbrella organizations, such as Heartbeat International and Care Net Inc., which support and influence the individual CPCs through training materials, fundraising support, or institutional oversight [21]. CPCs attract clients through offers of free medical services (e.g., urine pregnancy tests and fetal ultrasounds) and, conditional on clients’ continuation of the pregnancy, access to material goods such as diapers and baby clothes [22,23]. CPCs counsel pregnant clients on a limited range of pregnancy options, emphasizing the supposed dangers of abortion and encouraging clients to “choose parenting” or, if needed, pursue adoption [20]. When clients indicate intentions to seek an abortion, CPC staff use stigmatizing messages to try to dissuade them [24]. Research to date has documented false and misleading statements on CPC websites and in communication with clients [7–9,25]. CPC websites often minimize the efficacy of condoms [8] and inflate the health risks of hormonal contraception [26]. CPCs also draw a non-existent connection between abortion and the risk of health conditions such as breast cancer, infertility, and suicide [7,9,27].

### 1.3 The present study

We expand upon research that establishes the prevalence of misinformation on CPC websites by turning to training materials from parent organizations. Parent organizations have significant influence over CPCs, and a condition of affiliation with parent organizations often includes the use of approved training materials [28]. We pair content analysis of these training materials with qualitative interview data collected among staff members working at Ohio-based CPCs to document similarities between the health messages present in these widely disseminated training materials and the content of the misinformation shared by CPC staff members during the qualitative interviews. The data collection was part of a larger project to study how policy affects reproductive health and equity in Ohio, a setting marked by sustained efforts to restrict access to reproductive healthcare during the study period [29–31].

## 2. Methods

### 2.1 Overview

We used two data sources. First, we analyzed an existing corpus of qualitative interviews with staff members from CPCs in Ohio. The primary objective of the qualitative interviews was to better understand how CPC staff viewed their work and interactions with clients, and their path to working at the CPC [32]. The interviewer (author AK) identified herself to participants as a researcher and sociologist. Participants were adults and provided written consent. Using thematic coding, we identified inaccurate health information shared by staff in these interviews. We then compared these messages to health information in the training manuals from Heartbeat International (“The Love Approach Leader’s Guide 3^rd^ ed.”) and Care Net (“Compassion, Hope, & Help: A Training Curriculum for Pregnancy Decision Coaching Trainer’s Guide”) which, in combination, served as parent organizations for all CPCs included in the study. The Ohio State University Institutional Review Board approved the study.

### 2.2 Sample and recruitment

We identified 24 CPCs in Columbus, Cincinnati, Cleveland or in the 100-mile radius of Columbus. This included both urban and rural CPCs. Between April 21^st^, 2019, and August 30^th^, 2019, we contacted the executive director of each CPC and asked to interview a staff member. Ten staff from eight CPCs consented and were interviewed, using audio-recording, in April 2019 to August 2019. The eight CPCs included in this study were affiliated with at least one parent organization, consisting of Heartbeat International, Birthright International, and Care Net. One CPC was affiliated with two parent organizations. CPC affiliation was determined through website statements or interlinked resources between individual CPC websites and parent organizations (e.g., shared hotline numbers, website infrastructure or management provided by parent organizations, and statements of policies and procedures with boilerplate language dictated by parent organizations).*2.3 Analytic Approach*

We transcribed and anonymized the qualitative interview recordings. Two authors (EGRW and AK) independently coded the transcripts for health information using a flexible coding approach [33], and a third author (PG) appraised the medical veracity of the messages drawing on the medical literature and on her expertise as a board-certified obstetrician/gynecologist with subspecialty training in complex family planning. We rated health messages as “false” if they directly contradicted established science or standards of care. We rated health messages as “misleading” if they offered incomplete information or partially factual information that might lead patients to misunderstand their own risk profile.

We ordered training manuals directly from the Heartbeat International and Care Net websites; these two organizations provided oversight for all but one of the CPCs in our sample. We were unable to access the training manual of Birthright International because the parent organization is based outside the United States and does not provide training materials to individuals unaffiliated with a local chapter. The Heartbeat International and Care Net manuals contained material for new volunteers and an instructor manual with a script for training and commentary about the content of the materials. Both training courses are intended to be taught over a few weeks with a total of at least 23 hours of instruction. The training manuals contain content quizzes for the new volunteers to take as an assessment of their learning. The first author (EGRW) coded the manuals for health information. We compared the health information messages in both datasets (the interviews and the training manuals) to identify commonalities between the two sets of messages in topic and content. EGRW highlighted instances where the health information included in training materials aligned with or deviated from the health messages present in the interviews and health messages identified in previous research.

## 3. Results

We identified seven false or misleading health messages that appeared in both the manuals *and* the interviews, which we grouped into larger themes: health messages related to abortion, health messages related to fertility, and health messages related to contraception. The specific health messages are: Abortion is widely available at all times; Medication abortion can be reversed; Abortion causes PTSD; Abortion is a form of trauma; Conception is only possible during a specific, set window of time; Contraceptives are abortifacients; and Contraceptive use increases STI susceptibility (Table 1). In all but one case (medication abortion reversal), these health messages were present in *both* sets of training materials. In the follow section, we walk through each theme individually, highlighting how it was framed in both interviews and the training materials. In addition, we present information on the demographics of our interview participants and an incidental finding about broader dissemination that emerged during analysis.

**Table 1:** Health messages with examples from training manual and qualitative interviews.

Health Message	Training Manual Excerpt	Interview Excerpt	Fact Check
*Theme: Abortion*			
Abortion is widely available, at all times	“The United States is one of four nations where a woman can get an abortion through all nine months of pregnancy for any reason.”	“When you are talking about New York making the laws of you know full-term abortion and even after the baby’s born, if it wasn’t successful, let’s kill it anyhow. You know?”	False [34,35]
Medication abortion can be reversed	“A desperate woman who has started the mifepristone abortion procedure and wants to know whether there is anything that can be done to save her baby. If she has only taken the first drug but not the second drug, it may not be too late.”	“We had another gal who um, her parents had take her for an abortion, um, and [the abortion] procedure was started um, medically, it was started. And then she changed her mind… That’s our only client who has gone through, you know, starting an abortion, starting an adoption plan, and then decided to parent.”	False [36]
Abortion causes PTSD	“65% of US women experienced multiple symptoms of PTSD, which they attributed to their abortions.”	“The statistics show us that uh, some of the trauma is as bad as PTSD. Like... (sighs) we know what that is when we think about people that are coming back from war, and it’s the same thing as abortion”	False [37]
Abortion is a form of trauma	“In reality, these women who are subsequently identified as being traumatized severely have failed to reach a true state of closure with regard to their [abortion] experience.”	“We did [a workshop] on trauma and abortion, you know previous abortion and stuff and the mental health individuals in the area all came to that event to learn more about how abortion may affect trauma”	False [38]
*Theme: Fertility*			
Conception is only possible during a specific, set window of time	“… Since the egg only lives for 24 hours after it is released from the ovary, the amount of time that you can get pregnant each month is just a few days.”	“NFP [natural family planning] is literally like, I know for sure, based off of my temperature, based off of my discharge, based off of whatever, I know for sure, after tracking, that I am fertile from this moment to this moment. It’s 72 hours.”	Misleading [39]. Although the fertile window is limited, most individuals do not have the level of training or expertise necessary to accurately track fertility.
*Theme: Contraception*			
Contraceptives are abortifacients	“The MAP [morning after pill] (and other hormonal contraceptives) can actually cause the death of an embryo before implantation, and are thus abortifacients.”	“Well I don’t know them by name but some of them do, are abortifacients that will expel a fertilized egg”	False [40]
Contraceptive use increases STI susceptibility	“Not only do birth control devices and chemicals have negative health (and behavioral) consequences, recent scientific studies indicate that some also may lead to a higher likelihood of contracting an STI/STD.”	“I just said we don’t have birth control. Tell them the terrible STI things that could happen.”	Misleading [41]. Although those who use contraceptives report higher levels of STIs, this is due to increased sexual activity relative to those who do not use contraceptives.

### 3.1 Participant demographics

Participants held a variety of paid and unpaid volunteer positions in their organization: executive director (n = 7), ultrasound technician (n = 1), client services manager (n = 1), and social media manager (n = 1). Staff members ranged in age from 27 to 71 years and belonged to a variety of Christian denominations (Table 2). Staff members in general had spent a significant portion of their career associated with CPCs, with some describing work experience at multiple organizations and many disclosing more than a decade in the field. Interviewees were asked about their own training for their current and previous positions. Many had previously been involved with a CPC as a volunteer and some supervised or trained volunteers directly.

**Table 2:** Participant demographics (N = 10).

Demographic characteristics	No.
*Race*	
White	9
Multiracial	1
*Educational attainment*	
Some college or associate’s degree	4
Bachelor’s degree or higher	6
*Religious Identity*	
Catholic	3
Evangelical Christian	4
Protestant, non-Evangelical	3

### 3.2 Abortion misinformation

With regards to abortion misinformation, across interviews and training manuals, we found inaccurate messaging regarding the availability of abortion, misleading information about “abortion pill reversal,” and false statements emphasizing a connection between abortion and later mental health risk (see Kelly 2013 for more information about the emergence of “post abortion syndrome”) [42].

Our results illuminated a mirroring in the language used by staff members which often referenced the statistics and information provided in the institutional training materials. Some staff members alleged the availability of “abortion pill reversal” and stated that it is a service they would like to offer to their clients. As the social media manager for one CPC said: “I would really like to do the abortion pill reversal. I think that’d be amazing to have…I don’t know how it works. Just seems like magic, but it works.” This endorsement of “abortion pill reversal” echoed a statement, which appeared solely in the Care Net training materials, around offering this service to clients, “Pregnancy centers can expect to get a phone call or visit from a desperate woman who… wants to know whether there is anything that can be done to save her baby. If she has only taken the first drug (mifepristone) but not the second drug (misoprostol), it may not be too late.” No information is provided in the Care Net training materials about how abortion pill reversal would work.

### 3.3 Fertility and contraceptive misinformation

Both staff and training materials offered misleading information about the efficacy of fertility awareness-based methods. There were statements in the training materials about these methods being “100% effective” if used properly, while statements around the efficacy of condoms, in contrast, described the devices as “very ineffective” unless used perfectly. Training materials from Heartbeat International explicitly stated that “...condoms do not protect from STIs and STDs.” Training materials also alleged a connection between contraceptive use and infertility later in life, attributing STI risk (and associated infertility) to the use of contraceptives: “This misunderstanding of the gift of fertility has led to devastating effects on women. Ironically, infertility sometimes is the result of following the so-called reproductive health advice that promotes contraceptives, “safer sex,” and abortion as standard practices.” The training manual states that contraceptive methods “are actually unhealthy” for the people who use them, causing “negative health (and behavioral) consequences” and potentially leading “...to a higher likelihood of contracting an STI/STD.”

Slippage in terminology between abortion and contraception was common. We documented a framing of fertilization as the start of pregnancy rather than implantation of a zygote as the start of pregnancy. This effort allowed the umbrella organizations (and those trained with their materials) to argue that hormonal contraception, which manuals describe as preventing implantation through changes to the uterine lining, and emergency contraceptives are abortifacients. When asked about differences between contraceptives, one staff member stated, “I don’t know them by name but some of them are abortifacients that will expel a fertilized egg.” This framing was similarly exemplified by this excerpt from the Care Net Inc. training guide: emergency contraception “may also act to prevent implantation, which is an early form of abortion.”

### 3.4 Organizational identity and CPC messaging in the community

While not initially a part of our stated research objectives, over the course of our analysis we noticed regular reference to how and why CPCs might embrace a pseudomedical identity in their community. We have included those results here because they provide necessary context for the potential impact of concentrated false health messaging present at CPCs. CPC staff described disseminating false or misleading health messages to people in their communities, relying on a perception of organizational legitimacy to gain entry into educational and professional spaces. One staff member described an event the CPC hosted for local mental healthcare practitioners: “...individuals in the area all came to that event to learn more about how abortion may affect trauma on an individual.” The same center also hosted sexual education programs for students; CPC staff told the study interviewer that these programs emphasized that “sex within marriage is the ideal place to have sex.” Another CPC and its staff presented on teenage pregnancy at a local school, relaying aspects of their presentation to researchers by saying that they told students, “You may not survive a sexually transmitted disease. Yep, your pregnancy will change your life, whether you parent, whether you adopt, whatever you do. It will change you, but it won’t kill you.” This messaging was consistent with content from the training materials which overstated the risk of STIs and abortion and understated the risk of pregnancy in the US.

CPCs perceived themselves as medical spaces. When asked specifically whether she would describe the center she directed as a healthcare facility, the executive director of a CPC said, “Well, in the fact that we do pregnancy tests and ultrasounds, yes.” Later in the interview, the same CPC executive director disclosed that the organization does not have a doctor on staff. Similarly, staff referenced medical practitioners, potentially operating outside their scope of practice, as the source of the CPC’s medical oversight. A CPC executive director explained that the only medical professional with executive oversight in the organization was an oral surgeon on their board because they “needed someone from within the medical community.” Another CPC director who had no medical training took calls on their anti-abortion “option line” and said that he was able to talk to “abortion-vulnerable” women and have conversations dissuading them from abortion. He attributed his success to the fact that he “sounds like a doctor.” We note that perceiving, or presenting, the CPC as a medical space was a key arena where CPCs deviated from the instruction provided by parent organizations. Heartbeat International and Care Net Inc. both emphasized the importance of operating within the appropriate scope of practice, explicitly coaching trainees on the boundaries of legality when it comes to providing medical information to clients seeking help. For example, CareNet instructs volunteers to “use appropriate disclaimers” and to “avoid giving advice” in their guidelines for sharing medical information.

### 3.5 Summary of findings

**Fig 1:**
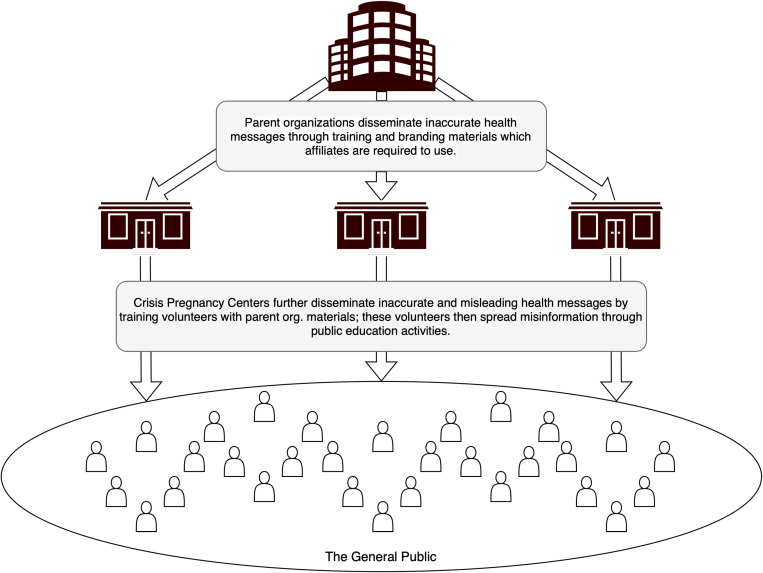
Diagram depicting the flow of false and misleading health messages from parent organizations to CPCs and then on to the general public.

Incorporating the incidental finding of organizational legitimacy allowed us to conceptualize the flow of misinformation from parent organizations to individual volunteers at CPCs and to the wider community (Fig 1). In addition, it underscored the oversized role that parent organizations play in disseminating reproductive health misinformation more broadly. Rather than attributing false and misleading statements to misinformed individuals or isolated bad actors, our findings indicate that institutional actors serve as high yield sources of medical misinformation.

## 4. Discussion

CPCs and the parent organizations that support and oversee them present, both in the statements of CPC staff members and in the written training materials of the parent organizations, internally consistent misinformation in the form of false and misleading health messages that they disseminate to CPC clients. We identified health messages in both staff interviews and training materials that are similar to those identified by previous researchers [7–9,25]. By associating misinformation at the local staff level to the same false and misleading messaging at the institutional level, we argue that the provision of inaccurate messages is not happenstance but rather is part of an intentional practice – on the part of the parent organizations – to spread misinformation that can influence people’s behavior.

Parent organizations make use of their organizational networks to broadly disseminate false and misleading information, often omitting relevant or more recent data in favor of misleading or outdated research. For example, in framing pregnancy and childbirth as safer than STIs, the training materials and staff members did not acknowledge that the US currently has the highest rate of maternal mortality among high income countries [43,44] or that maternal mortality is particularly elevated for Black women [43], a client population increasingly targeted by CPCs [45,46]. Similarly, CPC staff and training manuals referred to hormonal contraception as an “abortifacient” without acknowledging that their definition of “abortifacient” did not align with accepted medical terminology identifying the start of a pregnancy as implantation rather than fertilization [40]. CPC staff members then leverage the medically adjacent identity of CPCs to further disseminate false and misleading health information to the general population. As CPCs continue to expand their institutional footprint in the health care landscape [32,47], it could become increasingly common for members of the public, and particularly for pregnant people who visit CPCs, to access health misinformation through these organizations. Seperately, the growing ties between government and CPCs [32] will likely create an environment where reputable health organizaitons lose funding *and* where patients will face increasing difficulty differentiating between legitimate and illegitimate sources of health information.

### 4.1 Strengths and limitations

CPC staff participated in these interviews about CPC practices fully aware of the identity and affiliation of researchers. Staff were informed through consent processes that they were talking to a researcher-interviewer with training in sociology and reproductive health. We posit that the staff wholeheartedly endorsing misleading statements to the interviewer indicates how deeply held these beliefs are. Thus, a strength of the research was non-deceptive interviews with CPC staff members and inclusion of source materials from the parent organizations.

The primary limitation of this research is the small number of participants. The narratives presented in our findings, however, are not unique to these staff members; our findings corroborate previous research about misinformation from CPCs. In addition, the training manuals from Heartbeat International and Care Net are used by over 3,500 CPCs worldwide and at approximately 2,500 CPCs located in the US (including the CPCs that employed our participants). With 2,633 CPCs operating in the US in 2024 [48], Care Net and Heartbeat International training materials are used in nearly all US-based CPCs, demonstrating the universality of the false or misleading health information they disseminate. As such, our research complements existing work by demonstrating the broad endorsement of inaccurate health messages at the institutional and individual levels.

### 4.2 Conclusion

CPCs provide factually incorrect health messages directly to the clients who visit them. CPC parent organizations provide and legitimize this health information through codified training documents and misleading citations. As CPCs continue to benefit from state-sanctioned legitimacy [32], the provision of inaccurate health messages will increasingly affect the quality and types of care sought by people with reproductive health needs. Prior work has demonstrated that state-sanctioned dissemination of misinformation decreases accurate knowledge around abortion [19]. Future research is needed on the impact of CPCs to determine whether they similarly reduce the reproductive health knowledge of the communities they serve.
